# Antibiotic–Drug Interactions in the Intensive Care Unit: A Literature Review

**DOI:** 10.3390/antibiotics13060503

**Published:** 2024-05-29

**Authors:** Paweł Radkowski, Maria Derkaczew, Michał Mazuchowski, Annas Moussa, Katarzyna Podhorodecka, Justyna Dawidowska-Fidrych, Małgorzata Braczkowska-Skibińska, Daria Synia, Karol Śliwa, Marta Wiszpolska, Marta Majewska

**Affiliations:** 1 Department of Anaesthesiology and Intensive Care, Faculty of Medicine, Collegium Medicum, University of Warmia and Mazury in Olsztyn, 10-082 Olsztyn, Poland; pawel.radkowski@uwm.edu.pl (P.R.); m.derkaczew@gmail.com (M.D.); michal.mazuchowski@student.uwm.edu.pl (M.M.); katarzyna.podhorodecka@la-ligniere.ch (K.P.); malgorzata.braczkowska@uwm.edu.pl (M.B.-S.); daria.synia@student.uwm.edu.pl (D.S.); karol.sliwa@student.uwm.edu.pl (K.Ś.); 2 Hospital zum Heiligen Geist in Fritzlar, 34560 Fritzlar, Germany; amoussa@ekh-ks.de; 3 Department of Anaesthesiology and Intensive Care, Regional Specialist Hospital in Olsztyn, 10-561 Olsztyn, Poland; 4 Pro-Medica Hospital, 19-300 Ełk, Poland; dawidowska.justyna@gmail.com; 5 Department of Human Physiology and Pathophysiology, Faculty of Medicine, Collegium Medicum, University of Warmia and Mazury in Olsztyn, 10-082 Olsztyn, Poland; marta.wiszpolska@uwm.edu.pl

**Keywords:** antibiotic, antibiotic–drug interactions, drug–drug interactions, pharmacokinetics, intensive care units

## Abstract

Interactions between drugs are a common problem in Intensive Care Unit patients, as they mainly have a critical condition that often demands the administration of multiple drugs simultaneously. Antibiotics are among the most frequently used medications, as infectious diseases are often observed in ICU patients. In this review, the most important antibiotic–drug interactions, based on the pharmacokinetic and pharmacodynamic mechanisms, were gathered together and described. In particular, some of the most important interactions with main groups of antibacterial drugs were observed in patients simultaneously prescribed oral anticoagulants, NSAIDs, loop diuretics, and valproic acid. As a result, the activity of drugs can be increased or decreased, as dosage modification might be necessary. It should be noted that these crucial interactions can help predict and avoid negative consequences, leading to better patient recovery. Moreover, since there are other factors, such as fluid therapy or albumins, which may also modify the effectiveness of antibacterial therapy, it is important for anaesthesiologists to be aware of them.

## 1. Introduction

Antibiotics are one of the most commonly used groups of medications in Intensive Care Units (ICUs) since critically ill patients are more likely to be exposed to a large number of pathogens as well as have a weakened immune system, which is associated with impaired immune mechanisms that are derived from the critical state. Therefore, in the EPIC III (Extended Study on Prevalence of Infection in Intensive Care III) study, which was conducted in 2017 in ICUs around the world, it was revealed that 54% of patients had suspected or proven infection and 70% of them received at least one antibiotic for prophylactic or therapeutic purposes [1]. That is the reason why doctors who specialise in anaesthesiology and intensive care should be more aware of antibiotic–drug and antibiotic–antibiotic interactions that may appear in this particular group of patients.

Pharmacological treatment of ICU patients is aimed at the improvement and maintenance of their good health through polypharmacotherapy, which involves the administration of more than one drug. Therefore, it may increase the risk of drug–drug interactions (DDIs), which might even be life-threatening. However, most drug interactions only lead to a decrease or increase in the effectiveness of the pharmacotherapy or may cause unwanted side effects. 

It is crucial to recognize the clinical significance of potential DDIs in the ICU environment. This is because only half of the identified potential DDIs were deemed clinically relevant. Even after accounting for clinical significance, ICU patients often face heightened risks of adverse drug events including QT prolongation, bleeding, and neurological disturbances. In addition to clinical relevance, it is crucial to take into account factors such as the duration and timing of potential DDIs, along with contextual information [2].

The essential message for clinicians is to acknowledge, evaluate the balance between risks and benefits, and diligently monitor significant DDIs. Clinicians should aim to reduce the likelihood of pharmacodynamic potential DDIs by steering clear of drug combinations that have similar adverse effects. Additionally, they should work to minimize the risk of pharmacokinetic DDIs by avoiding combinations of drugs that are metabolized by the same cytochrome P-450 isoenzymes, and also avoiding combinations of these drugs with substances that inhibit or induce these enzymes. When necessary, clinicians should opt for medications that are unaffected by such inhibitors. Likewise, it is important to avoid combinations of drugs that are substrates of the P-glycoprotein efflux pump and/or its inhibitors, as well as inducers [3].

It should be noted that not all pharmacologic interactions are harmful. Some medications may be better absorbed if taken with a certain food, or their percentage in the bloodstream may increase if they are taken with other drugs that affect certain metabolic enzymes. Instead, on occasion, drugs cannot be co-administered intravenously because of their physical or chemical inconsistency. For example, in a recent investigation conducted in 2020, a multitude of intravenous incompatibilities came to light. The most common incompatible pair of drugs administered intravenously (IV) were meropenem and pantoprazole as continuous infusion and bolus dose, respectively. Less common interactions were observed while administering pantoprazole as a bolus dose with continuous infusion of clindamycin, piperecycline/tazobactam, or metronidazole [4]. To prevent the mentioned IV incompatibilities, different strategies were recommended, such as separate IV infusions in time and place, the use of in-line infusion filters, or multiple lumen catheters.

Serious and life-threatening drug interactions are not common, but they are often very predictable and should be a cause for concern involving the therapeutic team. Such patients must be monitored every time a new drug is added and sometimes even when discontinued, as it may affect serum concentrations of other drugs. There are also already listed groups of factors that determine the possibility of the occurrence of drug interactions and their severity, including the total number of drugs taken by the patient, drug class, patient age, liver and renal functions, diet, patient’s general health status and, lastly, the potential intersubject variability associated with the metabolic functions [5].

Information regarding drug interactions between antibiotics and medications used in the ICU remains limited in many areas of scientific inquiry. Gaps in the literature include medications still used in ICU settings as well as newly introduced drugs, which may not undergo extensive clinical trials due to the challenging conditions of critically ill patients, often at the end of life. Consequently, knowledge in this area relies heavily on observations and reports from other researchers. One increasingly utilized medication in our setting is dexmedetomidine, known for its sedative and calming effects. However, studies on its interactions with antibiotics, commonly administered in the ICU, are significantly lacking. There is also scarce information in the literature regarding the interactions of antibiotics with the commonly used analgesic in ICU patients—hydromorphone. Another intriguing topic is the possible interaction between antibiotics and inhalational anaesthetics such as isoflurane, but there are also still few reports in the literature describing this subject.

The current study aimed to thoroughly assess and review the literature on the theme of antibiotic use in the ICUs with a peculiar emphasis on potentially important interactions with other most commonly implemented medications, as this knowledge within the community of anaesthesiologists and intensive care practitioners may drastically improve their patients’ outcomes. In many cases, it may also be a life-saving remedy, as antibiotics are one of the most commonly used medications in ICUs, and considering their abundance, it is important to make the correct selection. To make this review, PubMed and Google Scholar databases were searched for the following inquiries: antibiotic interactions with other medications, antibiotics in the intensive care units or anaesthesiology and intensive therapy, antibiotic interactions in intensive care units or anaesthesiology and intensive therapy. 

## 2. Beta-Lactams

Beta-lactam antibiotics are one of the most frequently used groups of medications for the treatment of infectious diseases. As these drugs have common features from a biochemical perspective, they can be divided and classified into the following five groups: penicillins, cephalosporins, carbapenems, monobactams, and beta-lactamase inhibitors [6]. Their activity depends on the group to which they belong and may be quite broad, including Gram-positive and Gram-negative bacteria and anaerobic organisms. The mechanism by which beta-lactams exert their pharmacodynamic influence rests upon the inhibition of peptidoglycan layer biosynthesis within the bacterial cell wall, which underscores their bactericidal efficacy, particularly within the domain of Gram-positive microorganisms. Because of their frequent use, drug interactions might appear when a beta-lactam is administered along with another drug. When combined with oral anticoagulants, beta-lactams (especially penicillin) may increase the risk of haemorrhage. The combined therapy of Beta-lactams and allopurinol may increase the risk of skin rash, which should not be treated as a hypersensitive reaction to Beta-lactams [7]. They may also decrease the effectiveness of oral contraceptive drugs. Therefore, it is recommended to use other methods of contraception during antibiotic treatment with B-lactams and up to seven days after finishing [8]. 

### 2.1. Penicillins

Penicillins are one of the oldest groups of antibacterial drugs, discovered in 1928 [9], which nowadays are widely used to treat different bacterial infections, especially those caused by *Staphylococci* and *Streptococci* [10]. 

As with all beta-lactams, penicillins should not be combined with oral anticoagulants, as their interactions increase the risks of haemorrhage. It is not recommended to treat patients at the same time with penicillin and probenecid, as the latter can block the tubular secretion of penicillin G and lead to higher and longer levels of plasma concentrations of the drug. Probenecid may also decrease the volume of distribution of penicillin [11]. Considering the antagonistic effects of penicillins and erythromycin, sulphonamides and chloramphenicol should not be used simultaneously. While administered concurrently with methotrexate, penicillins may reduce renal secretion and result in increased systemic exposure. However, the recent guidelines for treatment of community-acquired pneumonia (CAP) recommend combination of macrolide and penicillin because of the broader coverage for the atypical organisms implicated in CAP and may contribute to antibacterial synergism. In the test designed by Lalitagauri M. Deshpande and Ronald N. Jones, the antagonism risk of erythromycin–penicillin interaction appeared to be very low and insignificant [12]. The improved survival rates are estimated at 20–50% in patients with severe CAP who were treated using a combination of macrolide and penicillin [13]. A combination of penicillin and non-steroidal anti-inflammatory drugs may lead to an increase in penicillin exposure [14]. When such drugs as aspirin, furosemide, indomethacin, and sulphonamides are used with penicillin, the half-life of the latter may be increased due to competitive inhibition of tubular secretion.

### 2.2. Cephalosporins

Cephalosporins belong to the group of beta-lactams, and they can be further divided into five generations considering their spectrum of activity against Gram-positive (G+) and Gram-negative (G−) bacteria. However, some authors question this well-known classification [6]. First-generation cephalosporins exhibit high activity against most G+ bacteria, including *cocci*: *Staphylococci* spp. and *Streptococci* spp. On the other hand, they demonstrate minimal activity against G− bacteria while having maximal coverage against *Proteus mirabilis*, *E. coli*, and *Klebsiella pneumoniae.* Medications belonging to the first-generation cephalosporins that are administered only parenterally include cefazolin, cefapirin, and cefalotin. Cefradine can be administered both orally as well as parenterally. Second-generation cephalosporins have higher activity against G− bacteria than G+ *cocci*. This category includes antibiotics that can be administered only parenterally, such as cefmetazole, cefotetan, and cefoxitin, while cefuroxime can be administered both orally and parenterally [6,15]. Higher activity towards Gram-negative bacteria is exhibited by third-generation cephalosporins. Therefore, they are very commonly used in ICUs to treat infections resistant to the lower classes of cephalosporins as well as sepsis of unknown origin. This particular group of antibiotics that are exclusively administered parenterally include cefotaxime, ceftazidime, and ceftriaxone. Third-generation cephalosporins can cross the blood–brain barrier, but only when given intravenously. Cefotaxime and ceftriaxone are especially potent when it comes to the ability to penetrate through the blood–brain barrier. Therefore, they are broadly used for treating bacterial infections of the central nervous system (CNS), such as meningitis. Amongst all third-generation cephalosporins, only ceftazidime is active against *Pseudomonas aeruginosa* [6,15,16]. Fourth-generation cephalosporins such as cefepime, cefiderocol, and cefpirome can cover infections caused by G− bacteria with a broader range of ability. They are all highly active against infections caused by *Pseudomonas aeruginosa* and can be administered only parenterally. However, their use in intensive care is reserved only for serious and multi-resistant infections. The further need to fight against the increasing resistance of many bacteria has been pushing researchers into finding new, potentially life-saving antibiotics. Fifth-generation cephalosporins, including ceftaroline, ceftolozane, and ceftobiprole, are generally used only intravenously. Ceftaroline is the only cephalosporin with the ability to cover against MRSA (methicillin-resistant Staphylococcus aureus). On the other hand, ceftolozane and ceftobiprole are active against *Pseudomonas aeruginosa* [6,15,16].

Antibiotic treatment with cephalosporins, especially from the III generation, may exacerbate the nephrotoxic effect of aminoglycosides and loop diuretics, which should be taken into consideration if patients are treated with this group of antibiotics. This DDI is related to the nephrotoxicity of each of these medications alone, and the combination of them can significantly potentiate adverse effects [17]. Special cautions need to be taken in patients with renal insufficiency covered by RRT (renal replacement therapy) when receiving treatment with ceftazidime or its conjunction with a beta-lactamase inhibitor (avibactam), as in this group of patients, ceftazidime-induced neurotoxicity can occur. Therefore, it is highly recommended for the therapeutic team to monitor ceftazidime serum concentrations [18].

### 2.3. Carbapenems

Carbapenems are another beta-lactam antibacterial drug class that has great potential considering their overall broad spectrum of antibacterial activity against G+ and G− aerobic and anaerobic bacteria and which are used only for severe infections as a reserved treatment against multiple-resistant pathogens [19]. Recent studies have shown that meropenem, imipenem, and ertapenem have approximately equal activity against most G+ and G− pathogens. In particular, imipenem, doripenem, and panipenem are mainly effective against G+ organisms, whereas biapenem, meropenem, ertapenem, and doripenem are moderately more helpful against G− microorganisms [20,21].

Because ICU patients usually have more serious illnesses and, as a result, higher risks of infectious diseases that are resistant to most antimicrobial drugs, carbapenems are the most frequently used antibiotics among those patients. Along with antimicrobials in ICU patients, other drugs are also prescribed to treat their severe conditions, which is why drug–drug interactions may appear. First of all, carbapenems could interact with valproic acid (VPA), decreasing valproate levels to subtherapeutic levels in the patient’s serum and inducing a higher risk of seizures [22,23]. Carbapenems’ decreasing effect on valproate levels can be explained by mechanisms at three sites: intestinal, liver, and blood. In the intestinal absorption: two potential mechanisms are observed: the inhibition of the intestinal transporter responsible for VPA absorption by carbapenem antibiotics and a reduction in b-glucuronidase production from enteric bacteria, which are killed by antibiotics. In the liver site, three potential mechanisms are described: a reduction in UDPGA levels by carbapenem antibiotics. UDPGA serves as a cofactor for UDP-glucuronosyltransferase (UGT)-mediated glucuronidation of VPA; the direct activation of UGT by carbapenem antibiotics, as observed following pre-incubation of human liver microsomes with these antibiotics; the inhibition of b-glucuronidase in the liver by carbapenem antibiotics, leading to a decrease in the amount of VPA liberated from VPA-Glu. In the distribution in blood, there is a mechanism in which carbapenem antibiotics inhibit transporters (Mrp4) responsible for effluxing VPA from erythrocytes to plasma, resulting in increased plasma VPA levels. The increased renal excretion of VPA as VPA-Glu depends on the elevation of VPA-Glu levels by UGT [24]

It also may lead to liver injury—in 1.52% of patients in valproate monotherapy, in 3.7% of patients receiving valproate with imipenem, and in 35.42% of patients receiving valproate combined with meropenem [25]. Therefore, it is quite important to include monitoring of valproate concentration during carbapenem therapy. To avoid interactions, other antimicrobial agents should be considered, if possible, in patients taking valproate for the treatment of epilepsy [26,27]. However, this drug–drug interaction might be useful in managing intentional or unintentional VPA overdose, as short-term meropenem dosing decreases serum levels of VPA and shows improvement in mental status shortly after administration [28]. Secondly, the combination of imipenem with amikacin could decrease the serum level of both drugs, so they should not be administered as a combined therapy [29].

### 2.4. Monobactams

Monobactams are the fourth out of the five groups of beta-lactam antibiotics that are used in ICU patients with complicated infections such as pneumonia or urinary tract infections and are effective in the treatment of Gram-negative bacteria and particularly useful in antipseudomonal activity [6]. 

Aztreonam is one of the most frequently used monobactams in ICU patients and is administered intravenously, which is why it is important to note some interactions that might appear while co-administered with other drugs in the ICU. In particular, co-administration of furosemide or probenecid can moderately increase the systemic exposure of aztreonam [11].

## 3. Fluoroquinolones

Fluoroquinolones are a class of antibiotic drugs used in numerous indications due to their high antibacterial activity and good tissue and cavity penetration [30]. Group I fluoroquinolones (e.g., Norfloxacin) are primarily active against G− organisms. With further development (group II: Ciprofloxacin/Ofloxacin, group III: levofloxacin, group IV: moxifloxacin), the spectrum of activity was expanded to include Gram-positive, anaerobic and atypical pathogens. For many years, ciprofloxacin was the drug of first choice for uncomplicated urinary tract infections due to the increased risk of serious side effects [31].

The oral absorption of all fluoroquinolones is significantly impaired when coadministered with aluminium- and magnesium-containing antacids and sucralfate, as well as with other polyvalent metal cations such as calcium and iron. Concomitant use of these agents, even when administered within several-hour intervals, should be avoided because it decreases fluoroquinolone serum levels [32]. However, this particular sort of drug–drug interaction in ICU patients is quite rare because all antibiotics are preferred to be administered intravenously. Enoxacin and ciprofloxacin impair the hepatic metabolism of theophylline and caffeine, leading to significantly increased serum concentrations. Ofloxacin and lomefloxacin have only minimal effects on xanthine metabolism. Ciprofloxacin can increase fluoroquinolones when administered with nonsteroidal anti-inflammatory agents and may lead to convulsions [33]. They may also prolong QTc interval in healthy patients, especially when combined with other drugs with the same mechanism of action, but it seems to be a drug interaction without clinical significance if administered at optimal doses [27]. Among this group of antibiotics, delafloxacin did not demonstrate any clinically significant impact on the QT/QTc interval in several studies [34,35]

According to the latest studies, fluoroquinolones and DOAC (direct oral anticoagulants) may moderately increase the risk of bleeding by increasing systemic levels of DOAC. Investigators Tatsuya Yagi and Buster Mannheimer specify that 26 out of 9783 patients (0.27%) had bleeding events in the concomitant use window and 0.6% in the extended window of 30 days [36].

## 4. Macrolides

Another frequently used class of antibiotics in ICU patients is macrolides because of their wide antibacterial activity [37]. The most important agents, azithromycin, erythromycin, and clarithromycin, are commonly used to treat pneumonia, tonsillitis, sinusitis, otitis media, uncomplicated skin infections, and sexually transmitted infections as they are active against such microorganisms as *Streptococcus* sp., *Staphylococcus* sp., *Haemophilus influenzae*, *Neisseria gonorrhoeae*, *Neisseria meningitides*, *Haemophilus influenzae*, and some intracellular pathogens such as *Chlamydia pneumoniae* and *Mycoplasma pneumoniae* that may cause atypical pneumonia [38]. They are also active against *Helicobacter pylori*, especially clarithromycin, which is used to treat Helicobacter-associated infections in standard triple therapy [39].

When prescribing macrolides, it is important to remember possible drug–drug interactions that may appear. First of all, colchicine combined with macrolides is more likely to induce heart or liver failure or even lead to death than drug combinations from other groups of antibiotics [40,41]. Co-administration of macrolides with digoxin may increase its serum level and lead to serious cardiac adverse effects. Digoxin serum levels should be monitored during combined therapy [42]. Additionally, macrolides may increase the serum level of carbamazepine, so its level should be monitored during therapy [43]. Furthermore, as well as carbapenems, concomitant use of erythromycin or clarithromycin and valproic acid may significantly increase valproate serum levels by inhibition of CYP450, which leads to valproate toxicity manifested by hypotension, bradycardia, CNS depression/encephalopathy, respiratory depression, cerebral oedema, metabolic acidosis, and can even progress to coma and death [44,45]. There was also an interaction revealed between clarithromycin and statins due to the inhibition of CYP3A4 and increased serum statin concentrations that cause rhabdomyolysis and other serious adverse drug reactions (ADR) of statins [46]. Moreover, macrolides, along with fluoroquinolones (as noted above), may also interact with DOAC and notably increase the statistically significant risk of haemorrhage [47].

## 5. Linezolid

The other antibacterial drug often used in ICU patients is linezolid, which has antibacterial activity against Gram+ bacteria and can penetrate the central nervous system due to its lipophilic quality. Due to its properties, linezolid is approved for the treatment of vancomycin-resistant enterococcal infections, pneumonia, and skin infections [48]. Apart from its antimicrobial activity, linezolid is also a reversible non-selective monoamine oxidase (MAO) inhibitor [49]. 

Such a mechanism of action is connected to many drug–drug adverse events with serotonin reuptake inhibitors, and other antidepressants, e.g., citalopram, escitalopram [50], methadone [51], sertraline [52], rasagiline [53], and fluoxetine [54], may lead to serotonin syndrome. However, in a cohort study led by Anthony D. Bai et al., it was revealed that concomitant use of linezolid and antidepressants did not significantly increase the risk of serotonin syndrome in older people. But even so, when prescribing those two drugs at the same time, it is crucial to remember their potential DDI [55].

Additional drug–drug combinations that lead to serotonin syndrome are linezolid and fentanyl. The mechanism of this interaction is not recognized but should be considered in patients treated in intensive care units [56].

Other linezolid interactions are connected with digoxin—Yulin et al. described a patient with increased serum level of linezolid and digoxin during pneumonia and heart failure treatment. To reach the therapeutic level of linezolid, its dose had to be decreased two times, and the digoxin dose had to be decreased four times. The mechanism of interaction between these drugs is unknown and requires serum level monitoring during treatment [57].

Continuous therapy of warfarin with added linezolid to treat infection leads to increased PT-INR from 1.62 ± 0.32 to 3.00 ± 0.83 four or five days after linezolid administration, which decreased to 1.26 ± 0.1 one week after the end of this combined therapy. In this case, PT-INR should be monitored during therapy [58].

It is not recommended to use linezolid with rifampicin, as it may reduce the linezolid serum concentration to a subtherapeutic level; however, it is a good combination for multi-drug resistant Gram+ bacteria, so co-therapy is possible but requires drug level monitoring [59,60].

In papers by Pai et al. and Lin et al. [61,62] clinically significant DDIs have also been documented for proton pump inhibitors, calcium channel blockers CCBs, and more recently, for cyclosporine. All these drugs inhibit P-glycoprotein and therefore increase linezolid concentrations. Cyclosporine was specifically identified as having the largest effect on this reduction in linezolid clearance.

## 6. Other Drug–Drug Interactions in ICU Patients

Except for major classes of antibacterial drugs and their interactions with other drugs administered simultaneously, which were mentioned above, other drug–drug interactions may occur in ICU patients, and due to their importance, they also deserve proper attention. For example, co-administration of co-trimoxazol with warfarin significantly increases the risk of gastrointestinal bleeding during therapy [63]. Due to this unwanted interaction, it is recommended to use other antibiotics in patients receiving warfarin than co-trimoxazole [64]. Combining aminoglycosides with loop diuretics, such as furosemide or torsemide, increases the risk of ototoxicity. This polytherapy should be avoided [64]. Vancomycin is usually used in the treatment of serious Gram+ bacterial infections. When administered along with piperacillin/tazobactam, vancomycin significantly increases the risk of nephrotoxicity; therefore, renal function should be monitored during therapy [65]. 

A new class of antimicrobial drugs (glycines) that are represented by tigecycline are widely used in ICU patients with complicated diseases because of their ability to overcome resistance to currently existent drugs, such as multi-resistant Gram-bacteria, especially vancomycin-resistant enterococci, penicillin-resistant *Streptococcus pneumoniae*, and methicillin-resistant *Streptococcus aureus* [66]. Although this drug is relatively new, there are some interactions registered that are significant to remember. Firstly, tigecycline is likely to interact with cyclosporine, and this is evidenced by the case report [67]. The mechanism of this process is not completely understood yet, but as a result, the concentration of cyclosporine is increased, which demands a reduction of its dosage [68]. Secondly, it was noted that tigecycline may interact with tacrolimus when administered simultaneously in several case reports. It is not certain but it is supposed that tigecycline may inhibit CYP 450 3A4 and lead to the growth of tacrolimus levels. This demands accurate monitoring while treating patients and a reduction of the dosage if necessary [69,70].

## 7. Prevention and Management of Antibiotic–Drug Interactions

The prevention and management of antibiotic–drug interactions in the ICU requires a multidisciplinary approach that includes pharmacists, physicians, and nurses. This approach comprises medication reconciliation, drug monitoring, and dose adjustments. Medication reconciliation involves identifying and resolving discrepancies between the medications that the patient is taking and the medications that are ordered in the ICU. This process is essential to avoid potential drug interactions. Drug monitoring involves measuring serum levels of medications with a narrow therapeutic window or a high risk of toxicity. For example, the serum levels of the antiarrhythmic drug amiodarone should be monitored in patients receiving clarithromycin, a potent inhibitor of cytochrome P450 enzymes. Dose adjustments may be necessary to avoid antibiotic–drug interactions. The dose of the antibiotic ciprofloxacin may need to be increased in patients receiving the proton pump [71].

In addition, an important part of the prevention of drug–drug interactions is to identify and assess the risks of DDI frequently used in ICU patients, which is based on rapidly changing pharmacokinetics that depend on age and correspondingly on renal excretion, hepatic metabolism, volume of distribution, and protein binding. It is also essential to be sure that drugs being used and administered simultaneously intravenously are not incompatible physically or chemically. 

## 8. Factors That Can Modify the Effectiveness of Antibiotic Therapy

Other treatments administered simultaneously include albumins, fluid therapy, pressor amines, diuretics, and vitamin C. The mechanisms of action of the mentioned factors are described in detail in Table 1.

## 9. Conclusions

The proper management of antimicrobial therapy in ICUs requires great attention, as it includes a wide range of different groups of medications, which naturally accounts for the abundance of pharmacologically important interactions. The most relevant interactions are summarised in Table 2, which could be used at patients’ bedside as a checklist for pharmacotherapy that needs to be followed. Following these indications and avoiding potential interactions prevents serious complications, which in some severe cases might lead to death. In addition, appropriate polypharmacotherapy is thought to accelerate the patient’s quick recovery by reducing the period of hospitalisation in the Intensive Care Units in a way that improves their quality of life. 

## 10. Future Directions

The majority of Intensive Care Unit patients receive numerous of drugs due to their severe or life-threatening illnesses to restore health. Thus, different drug–drug interactions might occur and worsen the patient’s condition. As new drugs constantly appear, more investigations should be performed to provide a better understanding of those complex interrelations and their mechanisms.

Considering the growing multidrug resistance of bacteria to practically all groups of antibiotics, which entails introducing completely new antibiotics to the market, it would be very useful to create a database in which all potential drug–drug interactions could be thoroughly described. To do that, further trials assessing the interactions between antibiotics and old as well as new generations of drugs need to be performed. Such comprehensive databases should be constantly updated so medical practitioners would be promptly informed about the latest knowledge. 

Various electronic databases are employed as a tool to assess potentially harmful DDIs by documenting their prevalence. In the study by Roblek et al. the most commonly used software was Micromedex^®^ Drug-Reax, which is known for having the highest sensitivity among all databases. Less frequently utilized were Drug Interactions Facts^®^, Lexi-Interact^®^, Pharmavista^®^, EpocratesRx^®^, MediQ^®^, and Drug Interaction Checker^®^. The primary drawbacks of new, local databases and spontaneous reporting systems include restricted accessibility, incomplete reports lacking information such as concurrent medications, under-reporting of adverse drug reactions, and a lack of data regarding user demographics and drug exposure trends. To address these challenges, web-based commercially accessible electronic databases were developed and are widely employed in clinical settings. The available drug interaction databases listed above diverge in terms of identification, categorization, and alignment with clinical assessment. Clinicians should still exercise caution when utilizing such tools [92].

The creation of global studies on larger amounts of ICU patients all over the world can help to reveal new dangerous drug incompatibilities in shorter terms. In virtue of such cooperation, an early identification will assist in designing the guidelines for optimal multidrug treatment. 

Future investigations in this branch can improve and provide optimized therapy for each patient, considering their features and needs, so that the side effects of drug–drug interactions can be diminished and the best treatment can be suggested and used for a better outcome.

## Figures and Tables

**Table 1:** Factors that can modify the effectiveness of antibacterial therapy.

	Albumins	Fluid Therapy	Pressor Amines	Diuretics	Vitamin C
Mechanism of action	Antibiotics may bind with proteins (especially albumins), as this causes a decrease in the free fraction of antibiotics that cannot exhibit any antibacterial properties.	Fluid therapy may alter antibiotic dosage due to increased volume of distribution (Vd), particularly for hydrophilic antibiotics.	They may change the distribution of hydrophilic antibiotics.	Concomitant use of diuretics, particularly furosemide, and antibiotics with renal elimination that cause Cmax to occur simultaneously leads to increased renal elimination of antibacterial drugs.	Causes a decrease in urine pH, which may cause an increase in renal elimination of antibiotics that are more alkaline.
References	[72,73]	[74,75,76]	[77,78]	[79]	[80,81]

**Table 2:** Interaction mechanism of antibiotics with medication.

Antibiotic	Co-Medication	Mechanism of Interaction	References
Penicillins	Oral anticoagulants	Increased risk of haemorrhages	[14,82]
	Probenecid	Blockage of tubular secretion of penicillin G and increased levels of plasma concentrations of the drug; decrease the volume of distribution of penicillin	[83]
	Methotrexate	Penicillins may reduce renal secretion and result in increased systemic exposure	[84]
	NSAIDs	Increase in penicillin exposure	[83]
	Aspirin, Furosemide, Indomethacin, Sulphonamides	The half-time of the penicillin may be increased due to competitive inhibition of tubular secretion	[14,83,85,86]
Cephalosporins	Aminoglycosides and loop-diuretics	Cephalosporins potentiate the nephrotoxic effects of aminoglycosides and loop-diuretics	[18,87]
Carbapenems	Valproic acid	Decrease in valproic acid serum level, liver injury	[22,23,25,26,27,28]
	Amikacin	Lower serum level of both antibiotics	[29]
Monobactams	Furosemide/Probenecid	Moderately increase the systemic exposure of aztreonam	[11]
Fluoroquinolones	NSAIDs	Fluoroquinolones—convulsion-inducing effects remain unclear, have been suggested to antagonize the inhibitory effect of gamma-aminobutyric acid or active the excitatory N-methyl-D-aspartate receptors of brain neurons NSAIDs—some in vitro studies have shown that some NSAIDs may enhance the inhibitory effect of fluroquinolones on gamma-aminobutyric acid receptors	[88,89]
	Metal ions	Decreased antibiotic serum level	[32]
	Drugs involving QTc	Prolonging QTc to serious arrhythmia	[33]
	DOAC	Increase systemic levels of DOAC (higher risk of bleeding)	[36]
Macrolide	Colchicine	Heart failure; liver failure; death	[40,41]
	Digoxin	Increased digoxin serum level	[42]
	Carbamazepine	Increased carbamazepine serum level	[43]
	Valproic acid	Increase valproate serum levels (valproate toxicity)	[44,45]
	Statins	Increased serum statin concentrations that cause rhabdomyolysis	[46]
	DOAC	Increase systemic levels of DOAC (higher risk of bleeding)	[47]
Linezolid	Selective Serotonin Reuptake Inhibitors (SSRI): Citalopram Escitalopram Sertraline Fluoxetine Methadone Fentanyl	Serotonin syndrome: PK—peak concentration, area under plasma concentration curve, volume of distribution (VD), and lipophilicity PD—binding affinity (Ki) and IC50 for serotonin reuptake transporter (SERT) and 5-HT2A	[50,51,52,53,54,55,56]
	Digoxin	Increased levels of both drugs	[57]
	Warfarin	Prolonged PT-INR during therapy	[58]
	Rifampicin	Induces of P-glycoprotein that increase clearance of linezolid and decrease linezolid plasma concentrations	[59,60,62]
Co-trimoksazol	Warfarin	Increased risk of gastrointestinal bleeding	[63,64,90]
Aminoglycosides	Loop diuretics	Ototoxicity: aminoglycosides—target outer hair cells at the basal turn of the cochlea before affecting the apical cells and inner hair cells loop diuretics—decrease blood flow to the inner ear	[64,91]
Vancomycin	Piperacillin-tazobactam	Nephrotoxicity/ Acute Kidney Injury: Piperacillin-tazobactam—interstitial nephritis Vancomycin—tubular toxicity, oxidative stress, cast formation	[65]
Tigecycline	Cyclosporine	Increased concentration of cyclosporine (reduction of its dosage)	[66,68]
	Tacrolimus	Growth of Tacrolimus levels	[69,70]

## Data Availability

Not applicable.

## Abbreviations

ADR	Adverse drug reactions
CNS	Central nervous system
DDIs	Drug–drug interactions
DOAC	Direct oral anticoagulants
EPIC III	Extended Study on Prevalence of Infection in Intensive Care III
ICU	Intensive care units
IV	Intravenously
MAO	Monoamine oxidase
MRSA	Methicillin-resistant Staphylococcus aureus
PD	Pharmacodynamic
PK	Pharmacokinetic
UGT	UDP-glucuronosyltransferase
RRT	Renal replacement therapy
VPA	Valproic acid
